# Presenting symptoms of cancer and stage at diagnosis: evidence from a cross-sectional, population-based study

**DOI:** 10.1016/S1470-2045(19)30595-9

**Published:** 2020-01

**Authors:** Minjoung Monica Koo, Ruth Swann, Sean McPhail, Gary A Abel, Lucy Elliss-Brookes, Greg P Rubin, Georgios Lyratzopoulos

**Affiliations:** a University College London, London, UK; b National Cancer Registration and Analysis Service, Public Health England, London, UK; c Cancer Research UK, London, UK; d University of Exeter Medical School, St Luke's Campus, Exeter, UK; e Institute of Health and Society, Newcastle University, Sir James Spence Institute, Royal Victoria Infirmary, Newcastle upon Tyne, UK

## Abstract

**Background:**

Early diagnosis interventions such as symptom awareness campaigns increasingly form part of global cancer control strategies. However, these strategies will have little impact in improving cancer outcomes if the targeted symptoms represent advanced stage of disease. Therefore, we aimed to examine associations between common presenting symptoms of cancer and stage at diagnosis.

**Methods:**

In this cross-sectional study, we analysed population-level data from the English National Cancer Diagnosis Audit 2014 for patients aged 25 years and older with one of 12 types of solid tumours (bladder, breast, colon, endometrial, laryngeal, lung, melanoma, oral or oropharyngeal, ovarian, prostate, rectal, and renal cancer). We considered 20 common presenting symptoms and examined their associations with stage at diagnosis (TNM stage IV *vs* stage I–III) using logistic regression. For each symptom, we estimated these associations when reported as a single presenting symptom and when reported together with other symptoms.

**Findings:**

We analysed data for 7997 patients. The proportion of patients diagnosed with stage IV cancer varied substantially by presenting symptom, from 1% (95% CI 1–3; eight of 584 patients) for abnormal mole to 80% (71–87; 84 of 105 patients) for neck lump. Three of the examined symptoms (neck lump, chest pain, and back pain) were consistently associated with increased odds of stage IV cancer, whether reported alone or with other symptoms, whereas the opposite was true for abnormal mole, breast lump, postmenopausal bleeding, and rectal bleeding. For 13 of the 20 symptoms (abnormal mole, breast lump, post-menopausal bleeding, rectal bleeding, lower urinary tract symptoms, haematuria, change in bowel habit, hoarseness, fatigue, abdominal pain, lower abdominal pain, weight loss, and the “any other symptom” category), more than 50% of patients were diagnosed at stages other than stage IV; for 19 of the 20 studied symptoms (all except for neck lump), more than a third of patients were diagnosed at stages other than stage IV.

**Interpretation:**

Despite specific presenting symptoms being more strongly associated with advanced stage at diagnosis than others, for most symptoms, large proportions of patients are diagnosed at stages other than stage IV. These findings provide support for early diagnosis interventions targeting common cancer symptoms, countering concerns that they might be simply expediting the detection of advanced stage disease.

**Funding:**

UK Department of Health's Policy Research Unit in Cancer Awareness, Screening and Early Diagnosis; and Cancer Research UK.

## Introduction

Globally, cancer control strategies increasingly encompass the early diagnosis of symptomatic cancer alongside primary prevention policies and screening programmes.^1^, ^2^ Several countries have introduced health system interventions that aim to expedite investigation and diagnosis of symptomatic individuals presenting in primary care, whereas public health education campaigns aimed at raising awareness of cancer symptoms are increasingly being used both in high-income countries and in low-income and middle-income countries (appendix p 2).^3^, ^4^, ^5^, ^6^, ^7^

By their nature, early diagnosis interventions focus on the presenting symptoms of cancer. If the selected symptoms predominantly represent advanced-stage disease, however, these initiatives might have little impact in improving cancer outcomes. Understanding associations between presenting symptoms of cancer and stage at diagnosis is therefore an important consideration.^2^

Existing evidence about associations between presenting symptoms and stage at diagnosis is scarce and confined to specific cancer sites,^8^, ^9^, ^10^, ^11^, ^12^, ^13^, ^14^, ^15^ overlooking the fact that some symptoms at presentation (particularly those of a non-specific nature) are shared among different types of cancer; for example, abdominal pain is a common symptom of colorectal, ovarian, and renal cancer.^16^ Furthermore, symptoms are typically examined as being either present or absent, without consideration of the possible additive or interactive effects of multiple symptoms.

In this study, we therefore aimed to examine associations between common presenting symptoms of cancer and stage at diagnosis, using data from a population-based incident cohort of patients with cancer.

**Research in context**

**Evidence before this study**

We searched PubMed for articles published between Jan 1, 1980, and Jun 17, 2019, using the search terms “presenting symptom” or “symptom” AND “cancer” AND “stage”, with additional hand-searching of reference lists of identified papers and relevant subject reviews. We identified 12 studies on single cancer sites (five on ovarian cancer, three on colon or colorectal cancer, one on lung cancer, one on anal cancer, one on pancreatic cancer, and one on renal cancer), of which three examined associations between presenting symptoms and stage, adjusting for possible confounders. Only one study (on patients with colorectal cancer) considered both single presenting symptoms and symptom combinations.

**Added value of this study**

These findings characterise associations between common presenting symptoms and stage at diagnosis in a population-based incident cohort of patients with different cancers. For 13 of the 20 studied symptoms, more than 50% of patients were diagnosed with cancer at stages other than stage IV, and for 19 symptoms (all except for neck lump) more than a third were diagnosed at a stage other than stage IV.

**Implications of all the available evidence**

Common presenting symptoms have variable associations with advanced stage at diagnosis, although for all symptoms analysed in this cross-sectional study we found that large proportions of patients are diagnosed at stages other than stage IV. Early diagnosis initiatives such as public health campaigns aimed at raising awareness of possible symptoms of cancer and clinical guidelines for the assessment and investigation of patients with symptoms have the potential to help detect cancer at a non-advanced stage.

## Methods

### Study design and participants

For this cross-sectional, population-based study we analysed data of patients included in the National Cancer Diagnosis Audit (NCDA) in England.^17^ As described previously, general practitioners (GPs) and other health-care professionals in participating general practices provided information about the diagnostic pathway of patients identified as having been diagnosed with a malignant neoplasm in 2014 by Public Health England's National Cancer Registration and Analysis Service (NCRAS). The data were collated by NCRAS under regulation 2 of the Health Service (Control of Patient Information) Regulations 2002 legislation. Ethical approval for this study was obtained by the London Hampstead Research Ethics Committee (REC reference: 8/LO/0377).

439 general practices (comprising approximately 5% of all practices in England) submitted data on 17 042 tumour records. The sex, age, and cancer site distribution of included patients was representative of the contemporary national incident cohort.^17^ Furthermore, participating practices were similar to non-participating practices with regard to their demographic case-mix, patient experience scores, and referral rates for suspected cancer, but served slightly larger registered populations.^17^

We restricted our study population to symptomatic adult patients aged 25 years and older, diagnosed with one of 12 solid tumours with a high degree (≥85%) of stage completeness (in descending order): endometrial (95% complete staging), lung, rectal, breast, melanoma, prostate, colon, renal, bladder, ovarian, oral or oropharyngeal, or laryngeal cancer (85% complete staging), representing 79% of incident cases of solid tumours in England in the study year 2014^18^ (see appendix p 3 for the list of excluded cancers).

### Procedures

Information about stage at diagnosis in the study population was available from NCRAS as TNM stage I–IV; we defined advanced stage as TNM stage IV (see below for alternative categorisation of stage at diagnosis).

Information about presenting symptoms (specified as symptoms noted at first presentation before diagnosis and referral) was provided by participating GPs based on health records using a list of 81 prespecified symptoms (in “yes” and “no” format). We examined 19 symptoms recorded in at least 50 patients: abnormal mole, abdominal pain, back pain, breast lump, chest infection, chest pain, change in bowel habit, cough, dyspnoea, fatigue, haematuria, haemoptysis, hoarseness, lower abdominal pain, lower urinary tract symptoms, neck lump, post-menopausal bleeding, rectal bleeding, and weight loss. All other symptoms (n=59) were considered together in a 20th “any other symptom” category (appendix p 4). Therefore, patients could have a single presenting symptom or multiple symptoms (one or more of the above symptoms in any combination).

### Statistical analysis

We estimated the proportion of patients diagnosed at stage IV by single or multiple symptom status for each of the 20 symptoms. Because patients with different cancers often present with the same symptom,^16^ we examined the cancer site case-mix of each presenting symptom (namely, the proportion of patients with different cancers that are diagnosed following presentation with a particular symptom) to aid interpretation of our findings (see appendix pp 5–6 for cancer site signatures of all 20 symptoms).

Subsequently, we examined patient-level associations between symptoms and stage at diagnosis using logistic regression (stage IV *vs* stages I–III). Ideally, we would have studied associations between every symptom combination (each pair, triplet, and so on) and stage at diagnosis, because the presence of additional symptoms could affect a given symptom's association with the outcome of interest; however, this approach was not feasible given the large number of symptom combinations relative to the sample size. Instead, we modelled each presenting symptom as a pair of binary variables: one denoting its presence or absence, and the other denoting its presence or absence when recorded with other symptoms. Since we did not examine patients without symptoms, the constant was constrained to 0, fixing the baseline odds of stage IV to 1.

For each symptom, two odds ratios (ORs) could be estimated from the above model for each symptom: “single” and “multiple”. The single OR represents the association of a given symptom with stage at diagnosis when seen alone, compared with change in bowel habit (used as the reference category, since this was the most common symptom in the study population). Furthermore, by adding the coefficients from the first and second binary variables for each symptom, we estimated a multiple OR for each symptom, which represents its association with cancer stage when seen together with one or more of the other 19 symptom categories, compared with patients with multiple symptoms other than the symptom of interest. An OR value of 1 implies that stage at diagnosis is no different between patients with and without the symptom of interest among those with multiple symptoms.

We also adjusted for age group (categorised as 25–49 years, 50–59 years, 60–69 years, 70–79 years, and ≥80 years), sex (male *vs* female), index of multiple deprivation income domain quintiles (from 1 to 5, with 1 being least deprived and 5 being most deprived), ethnicity (white, non-white, and missing), and cancer site (12 solid tumour sites, as described above). The events per variable criterion for sample size considerations was satisfactory.^19^

We did four sensitivity analyses. For the first, we repeated the main analysis by categorising advanced stage as stage III–IV, compared with stage I–II disease.

The second sensitivity analysis comprised an extreme case scenario for missing information about stage. Unlike with missing exposure variable data, a complete case analysis (CCA) where only outcome variable data are missing (as is the case for stage at diagnosis in our study) is unbiased assuming the missing at random mechanism. Multiple imputation can make the assumption of data missing at random more reasonable than it would be under CCA only if auxiliary variables that are absent from the analysis model are used in the imputation model;^20^ however, no such variables were available and so the imputation model would have been of no value. Therefore, we did an extreme case sensitivity analysis in which all patients with missing stage information were assumed to have stage IV cancer. Although this extreme scenario is unlikely to be realistic, we can be reasonably confident that the true bias in our findings will be lower than that illustrated with this analysis.^21^

The third sensitivity analysis involved repeating the main analysis by restricting it to patients who had a diagnostic interval (time from symptomatic presentation to diagnosis) of 0–60 days, since time to diagnosis could influence the association between presenting symptoms and stage.^22^

For the fourth sensitivity analysis, we adjusted for route to diagnosis as the association between route to cancer diagnosis (a patient's health-care utilisation pathway before diagnosis) and stage might also influence the association of interest.^23^ Therefore, the main analysis was repeated with further adjustment for route to diagnosis among patients with complete information about their diagnostic route.^24^ Specifically, we considered the following five diagnostic route categories: 2-week-wait referral (urgent referrals for suspected cancer from primary care to specialist hospital services in the UK), elective referral (routine, non-urgent referrals), emergency presentation, secondary care (both inpatient and outpatient) routes, and unknown route.

All analyses were done with Stata SE, version 15.1.

### Role of the funding source

The funders of the study had no role in study design, data collection, data analysis, data interpretation, or the writing of the report. The corresponding author had full access to all the data in the study and had final responsibility for the decision to submit for publication.

## Results

**Figure 1:**
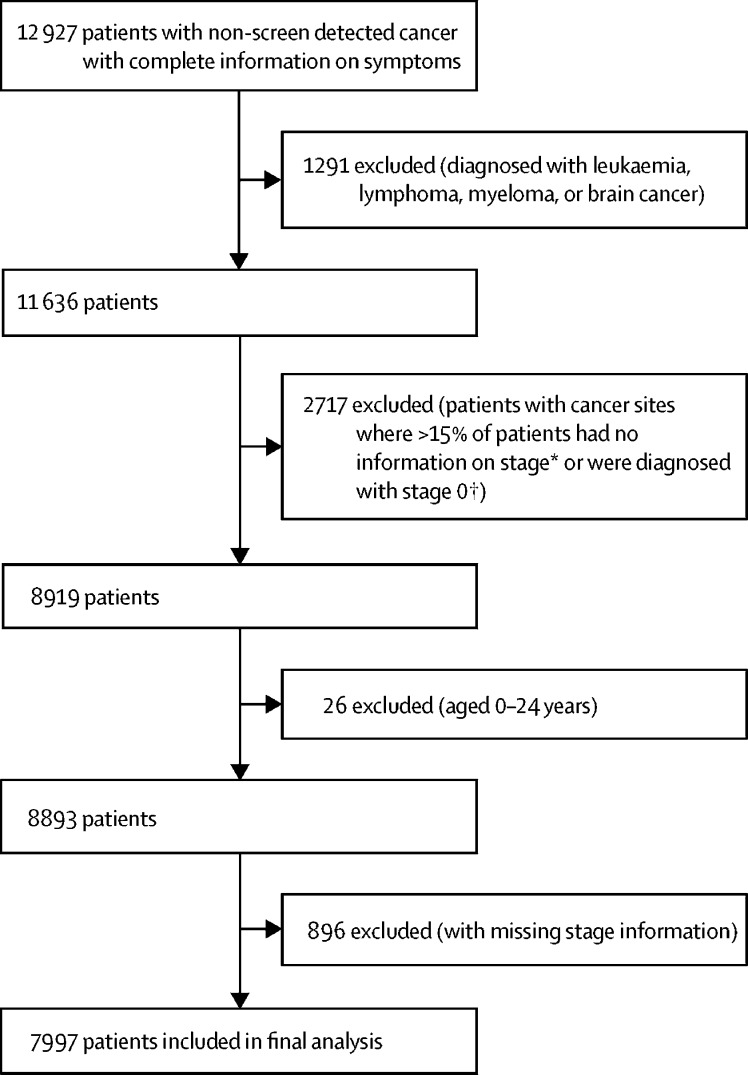
Study population *n=2707. †n=10.

**Figure 2:**
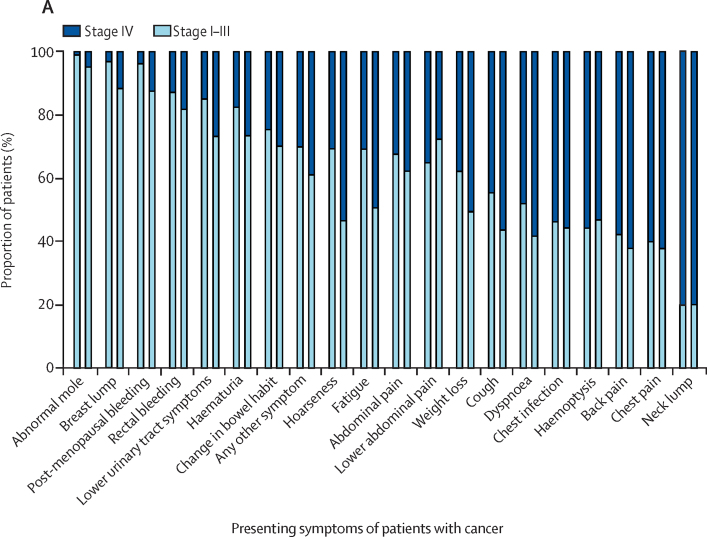
Presenting symptoms and proportions of patients with stage I–III and stage IV cancer The first bar of each pair for each symptom represents symptoms recorded alone (single symptoms), whereas the second bar of each pair represents symptoms recorded with other symptoms (multiple symptoms).

From an initial cohort of 12927 patients with non-screen detected cancers included in the NCDA 2014, we analysed data for 7997 patients diagnosed with solid tumours with high stage completeness (figure 1). Breast lump was the most common presenting symptom, reported in 1260 patients in our study sample, and neck lump was the least common, reported in 105 (table). A third of all patients (2685 of 7997; 34% [95% CI 33–35]) had two or more symptoms; symptoms varied in their likelihood of being seen alone or with other symptoms (figure 2).

**Table:** Observed proportions of stage IV cancer associated with 20 presenting symptoms (overall, as a single symptom, and as one of multiple symptoms) in patients diagnosed with one of 12 cancers (n=7997)

	**Overall**			**Symptoms reported alone**			**Symptoms reported with other symptoms**		
	Total (n)	Stage IV (n)	Stage IV (%; 95% CI)	Total (n)	Stage IV (n)	Stage IV (%; 95% CI)	Total (n)	Stage IV (n)	Stage IV (%; 95% CI)
Abnormal mole	584	8	1% (1–3)	564	7	1% (1–3)	20	1	5% (0–25)
Breast lump	1260	58	5% (4–6)	1074	36	3% (2–5)	186	22	12% (8–17)
Postmenopausal bleeding	292	17	6% (3–9)	229	9	4% (2–7)	63	8	13% (6–23)
Rectal bleeding	498	80	16% (13–20)	215	28	13% (9–18)	283	52	18% (14–23)
Lower urinary tract symptoms	1135	210	19% (16–21)	805	121	15% (13–18)	330	89	27% (22–32)
Haematuria	487	101	21% (17–25)	322	57	18% (14–22)	165	44	27% (20–34)
Change in bowel habit	819	236	29% (26–32)	186	46	25% (19–32)	633	190	30% (26–34)
Lower abdominal pain	285	83	29% (24–35)	51	18	35% (22–50)	234	65	28% (22–34)
Any other symptom	2433	873	36% (34–38)	876	265	30% (27–33)	1557	608	39% (37–42)
Abdominal pain	424	156	37% (32–42)	89	29	33% (23–43)	335	127	38% (33–43)
Hoarseness	124	51	41% (32–50)	68	21	31% (20–43)	56	30	54% (40–67)
Fatigue	365	170	47% (41–52)	58	18	31% (20–45)	307	152	50% (44–55)
Weight loss	584	287	49% (45–53)	71	27	38% (27–50)	513	260	51% (46–55)
Cough	672	361	54% (50–58)	161	72	45% (37–53)	511	289	57% (52–61)
Haemoptysis	179	97	54% (47–62)	59	33	56% (42–69)	120	64	53% (44–62)
Chest infection	317	176	56% (50–61)	63	34	54% (41–67)	254	142	56% (50–62)
Dyspnoea	513	289	56% (52–61)	108	52	48% (38–58)	405	237	59% (54–63)
Back pain	269	163	61% (54–66)	107	62	58% (48–67)	162	101	62% (54–70)
Chest pain	293	181	62% (56–67)	83	50	60% (49–71)	210	131	62% (55–69)
Neck lump	105	84	(80% (71–87)	65	52	80% (68–89)	40	32	80% (64–91)

Symptom rows are ordered by overall proportion of patients with stage IV cancer.

Some, typically localised, symptoms had relatively narrow cancer site signatures where the majority (>80%) of patients were diagnosed with the same cancer site, such as breast lump (breast cancer), abnormal mole (melanoma), post-menopausal bleeding (endometrial cancer), lower urinary tract symptoms (prostate cancer), and haemoptysis, dyspnoea, chest infection, chest pain, and cough (lung cancer). By contrast, less specific symptoms such as abdominal pain, change in bowel habit, back pain, fatigue, and weight loss had more diverse cancer site signatures (appendix pp 5–6).

The proportion of patients diagnosed at stage IV by symptom varied from 1% (95% CI 1–3; eight of 584 patients with an abnormal mole) to 80% (71–87; 84 of 105 patients with a neck lump; table, figure 2). For 13 of the 20 symptoms, more than 50% of patients were diagnosed with non-advanced stage cancer, while for all symptoms apart from neck lump, more than a third (at least 38%) were diagnosed at stages other than stage IV (table, figure 2).

**Figure 3:**
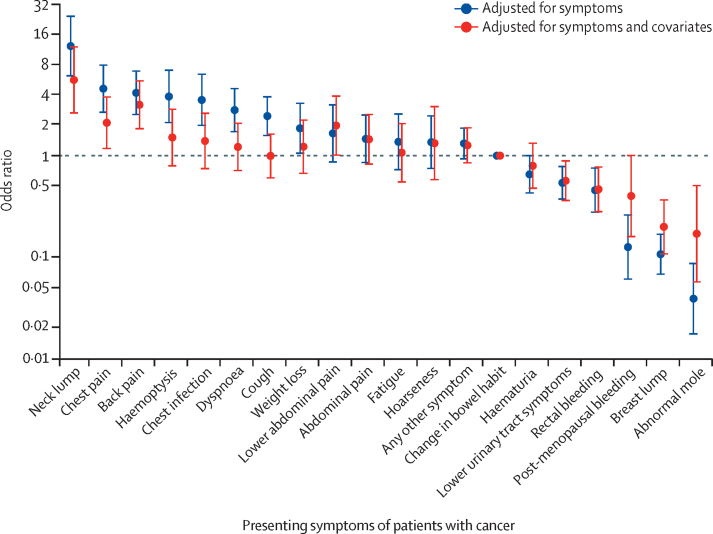
Odds ratios of stage IV disease by presenting symptoms seen alone Odds ratios of stage IV disease by symptom without adjustment (blue); and with adjustment for sex, age group, ethnicity, IMD quintile, and cancer diagnosis (red). Data shown for 7997 patients with one of 12 cancers. Error bars represent 95% CIs; the dashed line represents the value of the reference group (patients with change in bowel habit). For odds ratios of symptoms when reported with other symptoms, see appendix p 9.

The pattern of variation in symptom-specific associations with stage IV disease when reported alone (figure 3) was similar to the associations seen for symptoms when reported with other symptoms (both χ^2^ p<0·0010; appendix pp 7–9). Three symptoms (neck lump, chest pain, and back pain) were consistently associated with an increased odds of stage IV disease, whereas abnormal mole, breast lump, post-menopausal bleeding, or rectal bleeding were associated with lower odds of stage IV disease (appendix p 8).

Adjusting for patient characteristics and cancer site made little difference to the order of symptom-specific odds of stage IV disease for both single and multiple symptoms, although effect sizes were generally smaller (figure 3; appendix pp 7–10).

Additional sensitivity analyses examining differences in categorisation of stage (categorising advanced stage as stage III–IV), an extreme case scenario for missing information on stage, stratification by diagnostic interval (restricting the analysis to those with a diagnostic interval of 0–60 days), and additional adjustment for route to diagnosis also provided similar findings (appendix pp 11–20).

## Discussion

In our population-based cohort of patients with cancer, certain presenting symptoms, such as neck lump, had stronger associations with stage IV disease than others, but for most symptoms we found that large proportions of patients were diagnosed at stages other than stage IV. The relative order of symptom-specific associations was broadly comparable whether symptoms were seen alone or with other symptoms. Adjustment for confounders including cancer site made little difference to the overall pattern of associations between symptoms and stage at diagnosis.

With regard to associations between presenting symptoms and stage at diagnosis, direct comparisons with existing literature are challenging because published studies are cancer-site specific. Evidence from such studies provides an incomplete picture of associations between presenting symptoms and stage at diagnosis, because individuals who present with the same symptoms could be diagnosed with cancers of different sites. By contrast, in this study, we evaluated the presenting symptoms of patients with a range of common and rarer cancers and examined associations with and without adjustment for the case-mix of cancer sites in our study population. Nevertheless, in previous studies, among patients with colorectal cancer, rectal bleeding has been associated with earlier stage at diagnosis compared with abdominal pain and change in bowel habit,^9^, ^14^ whereas in patients with ovarian cancer, gastrointestinal symptoms (including abdominal pain, digestive bowel symptoms, and distension) have been more strongly associated with advanced stage cancer than gynaecological symptoms such as vaginal bleeding.^8^, ^10^

Except for one published study (which examined three symptoms and their combinations with stage^14^), the existing literature does not differentiate symptoms according to when they are reported on their own or when they are reported with other symptoms. By contrast, our study characterised associations between symptoms and stage, both when a symptom was reported alone and when it was reported together with other symptoms, which allowed us to adjust for potential interactions between all possible symptom combinations.

A key strength of our study is that it examined the association between presenting symptoms and stage in a well-characterised population-based incident cohort of patients with different cancers. The NCDA represents a unique combination of information provided by GPs and other health-care professionals based on clinical insight and judgment, with high-quality information about patient and tumour characteristics from the national cancer registry in England.^17^, ^25^

In addition to adjusting our findings by sociodemographic factors, we adjusted our results by cancer site. Although this adjustment did not appear to substantially alter the observed patterns of variation, the associations between symptoms and stage at diagnosis might differ in a population-based incident cohort with a different distribution of cancer sites.

Our study does have some potential limitations. As is the case for other studies based on clinical audits of cancer diagnosis,^26^, ^27^, ^28^ elicitation and recording of symptoms during the index consultation, and subsequent extraction of information from primary care records, might be incomplete and prone to bias.^29^ Nevertheless, alternative approaches based on self-reported data from individuals diagnosed with cancer are susceptible to under-representation of patients with a poor prognosis.^30^ With either method, it is possible that some of the recorded symptoms might relate to concomitant chronic illness, especially in patients with multiple symptoms. However, the observed distribution of cancer sites among patients with specific symptoms concord with previous knowledge about the presenting symptoms of each cancer site. This observation provides a strong indication that the recorded information about presenting symptoms chiefly relates to the subsequently diagnosed cancer site rather than other conditions.

The study population represents around four-fifths of patients diagnosed with solid tumours in England during 2014.^18^ Among the cancer sites included in the study, a small proportion of patients with missing stage information were excluded from analyses, variably by cancer site. However, sensitivity analyses, which would demonstrate the effect of maximum possible bias arising from missing data (ie, by assigning patients with missing stage information to stage IV) provided similar findings to our main analysis.

Our findings refute concerns that early diagnosis interventions centred on common presenting symptoms of cancer would typically expedite the diagnosis of individuals with stage IV disease. Rather, they indicate that a substantial proportion of patients with these symptoms are diagnosed with non-advanced disease, which is associated with potentially good prognosis. This finding was the case even for patients with symptoms most strongly associated with advanced stage in our study, and for symptoms often considered indicative of advanced disease, such as weight loss.

Furthermore, evaluation of the effect of single and multiple symptoms separately indicates that the presence of multiple symptoms is a poor predictor of stage IV disease. The nature of the symptom appears to be more important than the number of reported symptoms. This observation is reassuring, given that public health education campaigns typically do not focus on symptom combinations.

Symptom awareness and appraisal by patients and doctors is an important determinant of timely presentation and investigation^31^, ^32^, ^33^, ^34^ but the optimal design of early diagnosis interventions aimed at earlier recognition of possible cancer symptoms by members of the public and health-care professionals remains unclear. Alongside considerations such as cancer site incidence, psychosocial barriers to presentation, and the predictive value of symptoms, evidence about the associations between presenting symptoms and stage at diagnosis can help to guide the design of early diagnosis interventions. Our findings provide support for such interventions, and counter concerns that they simply expedite the detection of advanced-stage disease.
