# Supplementary material for: Presenting symptoms of cancer and stage at diagnosis: evidence from a cross-sectional, population-based study
Source: Lancet Oncol. 2020 Jan;21(1):73–9. doi: 10.1016/S1470-2045(19)30595-9 (PMC6941215; doi:10.1016/S1470-2045(19)30595-9)
Supplement: Supplementary appendix [file mmc1.pdf]

THE LANCET
Oncology

Supplementary appendix

This appendix formed part of the original submission and has been peer reviewed. We post it as supplied by the authors.

Supplement to: Koo MM, Swann R, McPhail S, et al. Presenting symptoms of cancer and stage at diagnosis: evidence from a cross-sectional, population-based study. *Lancet Oncol* 2019; published online Nov 5. http://dx.doi.org/10.1016/S1470-2045(19)30595-9.

Table of contents:

Examples of cancer symptom awareness campaigns in a range of high-income and low-middle income countries 2

Percentage of patients with missing information on stage at diagnosis by cancer site 3

The 20 defined symptoms of interest as derived from those described in the National Cancer Diagnosis Audit (NCDA) 4

The cancer site signatures of the 20 examined presenting symptoms of cancer 5

Two-dimensional heat map of cancer site signatures of the 20 examined presenting symptoms 6

Proportions and odds ratios of diagnosis at stage IV associated with 20 presenting symptoms when recorded alone 7

Proportions and odds ratios of diagnosis at stage IV associated with 20 presenting symptoms when recorded with other symptoms 8

Figure of odds ratios of stage IV disease by presenting symptoms reported with other symptoms 9

Rank order of symptom-specific ORs 10

Alternative parameterisation of advanced stage category as stage III–IV 11

Extreme case scenario for missing information on stage 13

Restricting analysis to patients who had a diagnostic interval (DI) of 0–60 days 15

Adjustment for route to diagnosis 18

Examples of cancer symptom awareness campaigns in a range of high-income and low-middle income countries

These include cancer symptom awareness campaigns in the United Kingdom (Public Health England, 2019), United States (Centers for Disease Control and Prevention, 2018), Ghana (AfrOx, 2019), Australia (Australian Cancer Council, 2019), and Malaysia (Be Cancer Alert collaboration, 2018).

[Images omitted due to potential copyright issues]

Sources:
https://www.nhs.uk/be-clear-on-cancer/
https://www.cdc.gov/cancer/knowledge/index.htm
http://www.afrox.org/55/cancer-prevention-posters-for-africa
https://www.findcancerearly.com.au/
http://www.becanceralert.com/

Percentage of patients with missing information on stage at diagnosis by cancer site

We restricted our study population to symptomatic adult patients diagnosed with one of 12 solid tumours with >85% completeness of information on stage at diagnosis (denoted by the shaded cells in the table below). Patients diagnosed with leukaemia (C91–95), lymphoma (C81–83, C85), multiple myeloma (C90), or brain cancer (C71) were excluded *a priori* (see Figure 2 in main text).

**Cancer**	**ICD-10 code**	**Total N**	**Stage missingness N (%)**
Endometrial	C54–55	371	20 (5%)
Lung	C33–34	1715	121 (7%)
Rectal	C19–20	520	41 (8%)
Melanoma	C43	750	76 (10%)
Prostate	C61	1577	168 (11%)
Breast	C50	1604	175 (11%)
Colon	C18	957	122 (13%)
Ovarian	C56	297	39 (13%)
Oral/oropharyngeal	C01–06, C09, C10	245	33 (13%)
Bladder	C67	423	57 (13%)
Renal	C64	370	50 (14%)
Laryngeal	C32	100	15 (15%)
Oesophageal	C15	394	79 (20%)
Stomach	C16	251	55 (22%)
Pancreatic	C25	412	108 (26%)
Testicular	C62	118	32 (27%)
Gallbladder	C23	40	11 (28%)
Thyroid	C73	118	49 (42%)
Small Intestine	C17	65	27 (42%)
Cervical	C53	70	34 (49%)
Other	*	649	339 (52%)
Mesothelioma	C48	34	20 (59%)
Liver	C22	182	108 (59%)
Vulval	C51	51	38 (75%)
CUP	C77–80	323	323 (100%)
Total	-	11636	2140 (18%)

CUP: cancer of unknown primary
*all other malignant cancer registrations beyond those specified

The 20 defined symptoms of interest as derived from those described in the National Cancer Diagnosis Audit (NCDA)

Symptom construct	Symptom(s) as originally described in NCDA
Breast lump	Breast lump/mass
Lower urinary tract symptoms (LUTS)	Lower urinary tract symptoms
Change in bowel habit (CIBH)	Change in bowel habit
	Constipation
	Diarrhoea
Cough	Cough
Weight loss	Weight loss
Skin lesion	Pigmented skin lesion
	Non-pigmented skin lesion
Dyspnoea	Dyspnoea
Rectal bleeding	Rectal bleeding
Haematuria	Haematuria
Abdominal pain	Abdominal pain NOS
Fatigue	Fatigue
Lower abdominal pain	Lower abdominal pain
Chest pain	Chest pain
Chest infection	Chest infection
Post-menopausal bleeding (PMB)	Post-menopausal bleeding
Back pain	Back pain
Hoarseness	Hoarseness
Haemoptysis	Haemoptysis
Neck lump	Neck lump/mass
Any other symptom	Remaining 59 symptoms recorded in NCDA

NOS: not otherwise specified

The cancer site signatures of the 20 examined presenting symptoms of cancer

As patients with different cancers often present with the same symptom[1], we examined the cancer site case-mix of each presenting symptom (namely the percentage of patients with different cancers diagnosed following presentation with a particular symptom) to aid interpretation of our findings.

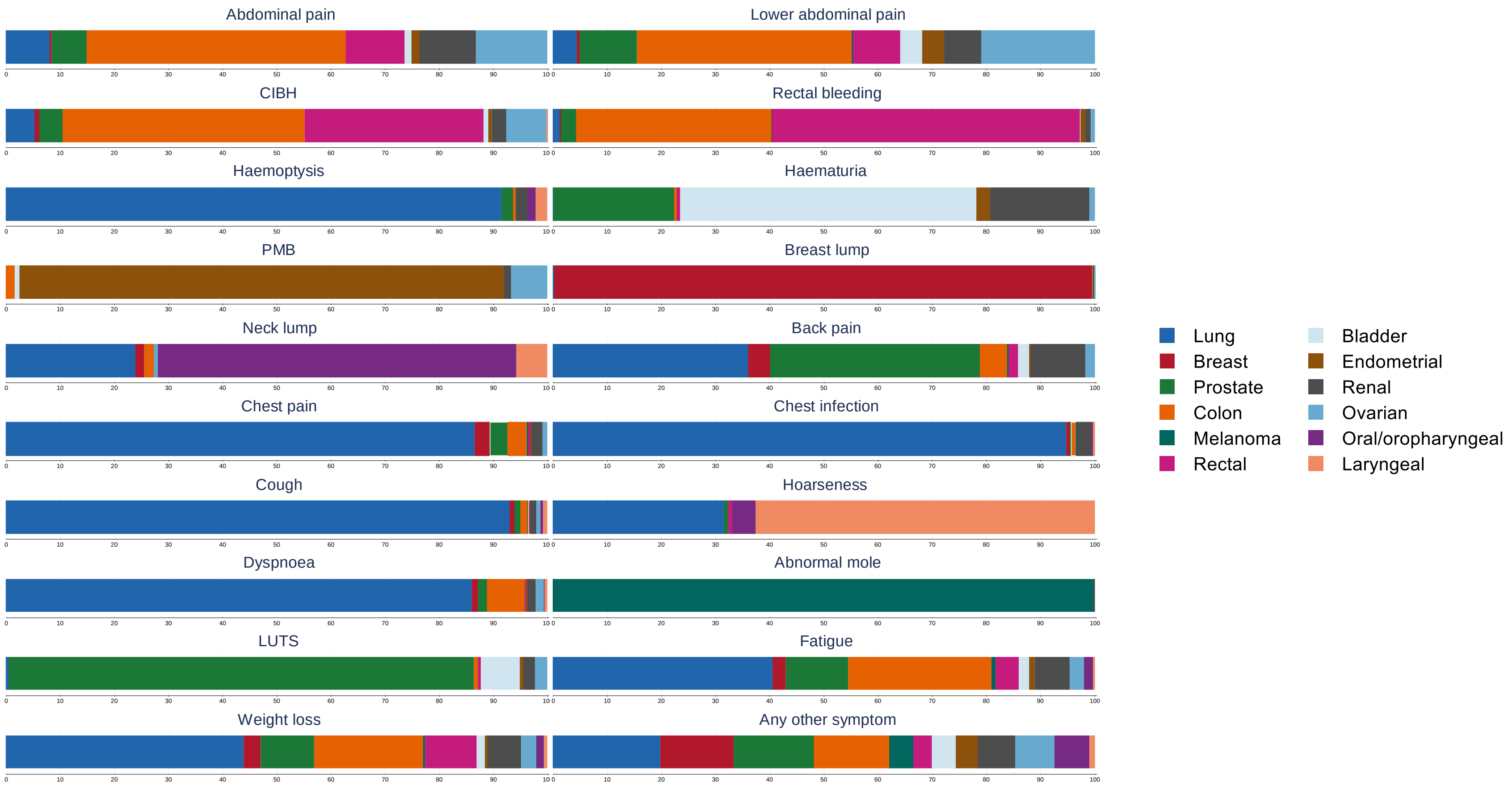

CIBH: change in bowel habit; LUTS: lower urinary tract symptoms; PMB: post-menopausal bleeding

[1] Koo MM, Hamilton W, Walter FM, Rubin GP, Lyratzopoulos G. Symptom Signatures and Diagnostic Timeliness in Cancer Patients: A Review of Current Evidence. Neoplasia 2017; 20: 165–74.

Two-dimensional heat map of cancer site signatures of the 20 examined presenting symptoms

Percentages are calculated by row; e.g. 55% of cancer patients who presented with haematuria were subsequently diagnosed with bladder cancer, 22% with prostate cancer, and 18% with renal cancer etc. Darker colours indicate higher percentages (relative ranking of frequency by column).

	Bladder	Breast	Colon	Endometrial	Laryngeal	Lung	Melanoma	Oral/ Oropharyngeal	Ovarian	Prostate	Rectal	Renal
Abnormal mole	–	–	–	–	–	–	100%	–	–	–	–	0%
Breast lump	–	99%	0%	0%	–	0%	0%	–	0%	–	–	–
PMB	1%	–	2%	89%	–	–	–	–	7%	–	–	1%
Rectal bleeding	0%	0%	36%	1%	–	1%	0%	–	1%	3%	57%	1%
Haematuria	55%	–	1%	3%	–	0%	–	–	1%	22%	1%	18%
LUTS	7%	0%	1%	1%	–	0%	–	–	2%	86%	0%	2%
Lower abdominal pain	4%	1%	40%	4%	–	4%	0%	–	21%	11%	9%	7%
CIBH	1%	1%	45%	1%	0%	5%	–	–	8%	4%	33%	3%
Abdominal pain	1%	0%	48%	1%	–	8%	–	–	13%	6%	11%	10%
Any other symptom	4%	14%	14%	4%	1%	20%	4%	6%	7%	15%	3%	7%
Fatigue	2%	2%	26%	1%	0%	41%	1%	2%	3%	12%	4%	6%
Weight loss	2%	3%	20%	0%	1%	44%	0%	2%	3%	10%	10%	6%
Haemoptysis	–	–	1%	–	2%	91%	–	2%	–	2%	–	2%
Hoarseness	–	–	–	–	63%	32%	–	4%	–	1%	1%	–
Chest infection	–	1%	1%	–	0%	95%	0%	0%	–	0%	–	3%
Cough	–	1%	1%	0%	1%	93%	0%	0%	1%	1%	0%	1%
Dyspnoea	–	1%	7%	0%	1%	86%	–	0%	1%	2%	0%	2%
Back pain	–	3%	4%	0%	–	86%	0%	–	1%	3%	0%	2%
Chest pain	–	3%	4%	0%	–	86%	0%	–	1%	3%	0%	2%
Neck lump	–	2%	2%	–	6%	24%	–	66%	1%	–	–	–

CIBH: change in bowel habit; LUTS: lower urinary tract symptoms; PMB: post-menopausal bleeding

Proportions and odds ratios of diagnosis at stage IV associated with 20 presenting symptoms when recorded alone

Reference group = patients with change in bowel habit alone.

Symptom (seen alone)	N (%) with stage IV	Unadjusted OR (95% CI)[1]	Adjusted OR (95% CI)[2]
Abnormal mole	7 (1%)	0·04 (0·02–0·09)	0·17 (0·06–0·51)
Breast lump	36 (3%)	0·11 (0·07–0·17)	0·20 (0·11–0·37)
PMB	9 (4%)	0·12 (0·06–0·26)	0·40 (0·16–1·02)
Rectal bleeding	28 (13%)	0·46 (0·27–0·77)	0·46 (0·27–0·78)
LUTS	121 (15%)	0·54 (0·37–0·79)	0·56 (0·35–0·90)
Haematuria	57 (18%)	0·65 (0·42–1·02)	0·79 (0·47–1·35)
CIBH	46 (25%)	Ref	Ref
Lower abdominal pain	18 (35%)	1·66 (0·85–3·22)	1·98 (1·00–3·94)
Any other symptom	265 (30%)	1·32 (0·92–1·90)	1·27 (0·84–1·92)
Abdominal pain	29 (33%)	1·47 (0·84–2·56)	1·45 (0·81–2·59)
Hoarseness	21 (31%)	1·36 (0·74–2·51)	1·33 (0·57–3·10)
Fatigue	18 (31%)	1·37 (0·72–2·62)	1·07 (0·54–2·10)
Weight loss	27 (38%)	1·87 (1·04–3·35)	1·23 (0·66–2·28)
Cough	72 (45%)	2·46 (1·56–3·88)	0·99 (0·59–1·65)
Haemoptysis	33 (56%)	3·86 (2·09–7·13)	1·51 (0·78–2·92)
Chest infection	34 (54%)	3·57 (1·96–6·48)	1·40 (0·73–2·66)
Dyspnoea	52 (48%)	2·83 (1·71–4·68)	1·22 (0·70–2·12)
Back pain	62 (58%)	4·19 (2·52–6·97)	3·19 (1·82–5·59)
Chest pain	50 (60%)	4·61 (2·66–8·00)	2·12 (1·16–3·86)
Neck lump	52 (80%)	12·17 (6·09–24·35)	5·62 (2·61–12·13)
Joint Wald test	-	p<0·0010	p<0·0010

CIBH: change in bowel habit; LUTS: lower urinary tract symptoms; PMB: post-menopausal bleeding

[1] adjusted for symptoms

[2]adjusted for symptoms, sex, age group, ethnicity, IMD quintile, cancer diagnosis

Proportions and odds ratios of diagnosis at stage IV associated with 20 presenting symptoms when recorded with other symptoms

Reference group = patients with multiple symptoms other than the given symptom of interest. Odds ratio values of 1 imply that when other symptoms are seen in combination with the symptom of interest this makes no difference to its association with stage at diagnosis.

**Symptom (seen with other symptoms)**	**N (%) with stage IV**	**Unadjusted OR (95% CI)[1]**	**Adjusted OR (95% CI)[2]**
Abnormal mole	1 (5%)	0·06 (0·01–0·44)	0·44 (0·05–3·77)
Breast lump	22 (12%)	0·14 (0·09–0·22)	0·45 (0·25–0·80)
PMB	8 (13%)	0·18 (0·08–0·38)	0·79 (0·33–1·88)
Rectal bleeding	52 (18%)	0·33 (0·23–0·46)	0·50 (0·35–0·74)
LUTS	89 (27%)	0·44 (0·34–0·58)	0·77 (0·57–1·05)
Haematuria	44 (27%)	0·53 (0·36–0·76)	0·98 (0·65–1·47)
CIBH	190 (30%)	0·67 (0·54–0·83)	1·10 (0·85–1·41)
Lower abdominal pain	65 (28%)	0·59 (0·43–0·80)	0·91 (0·66–1·26)
Any other symptom	608 (39%)	0·94 (0·82–1·08)	1·55 (1·31–1·85)
Abdominal pain	127 (38%)	0·89 (0·69–1·14)	1·37 (1·04–1·79)
Hoarseness	30 (54%)	0·94 (0·54–1·62)	1·32 (0·71–2·44)
Fatigue	152 (50%)	1·03 (0·80–1·32)	1·20 (0·92–1·56)
Weight loss	260 (51%)	1·27 (1·04–1·55)	1·34 (1·08–1·65)
Cough	289 (57%)	1·09 (0·86–1·37)	1·03 (0·80–1·31)
Haemoptysis	64 (53%)	0·91 (0·62–1·34)	0·97 (0·66–1·44)
Chest infection	142 (56%)	1·00 (0·75–1·34)	0·97 (0·72–1·30)
Dyspnoea	237 (59%)	1·25 (0·99–1·58)	1·28 (1·00–1·64)
Back pain	101 (62%)	2·05 (1·46–2·87)	2·76 (1·93–3·94)
Chest pain	131 (62%)	1·42 (1·05–1·92)	1·45 (1·06–1·98)
Neck lump	32 (80%)	3·91 (1·79–8·56)	3·55 (1·56–8·08)
Joint Wald test	-	p<0·0010	p<0·0010

CIBH: change in bowel habit; LUTS: lower urinary tract symptoms; PMB: post-menopausal bleeding

[1] adjusted for symptoms

[2]adjusted for symptoms, sex, age group, ethnicity, IMD quintile, cancer diagnosis

Figure of odds ratios of stage IV disease by presenting symptoms reported with other symptoms

Odds ratios of stage IV disease by symptom without adjustment (blue); and with adjustment for sex, age group, ethnicity, IMD quintile, and cancer diagnosis (orange) (n=7997 patients with one of 12 cancers). Error bars represent 95% CIs; the dashed line represents the value of the reference group (patients with change in bowel habit).

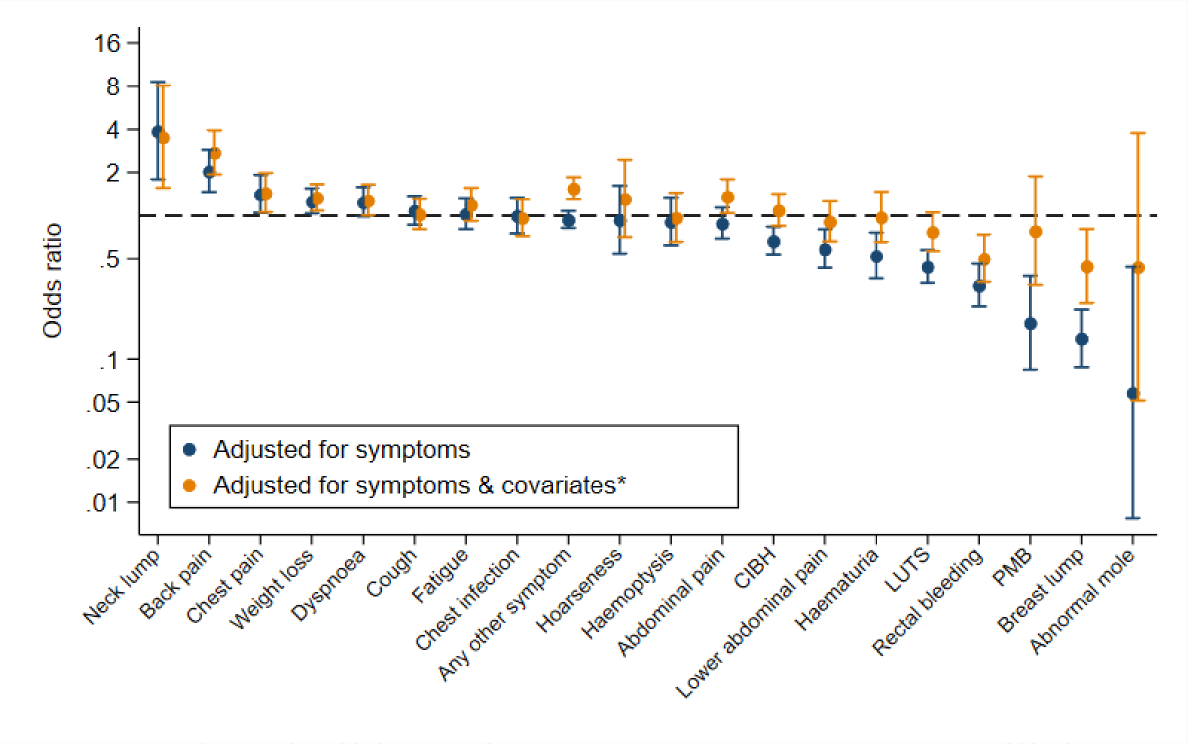

CIBH: change in bowel habit; LUTS: lower urinary tract symptoms; PMB: post-menopausal bleeding
**Sex, age group, ethnicity, IMD quintile, cancer diagnosis*

Rank order of symptom-specific ORs

Ladder diagram indicating change in relative position of the 20 examined symptom categories in association with diagnosis at stage IV when reported alone, versus when reported with other symptoms (based on ORs adjusted for symptoms, sex, age group, ethnicity, IMD quintile, and cancer diagnosis).

**Rank of adjusted ORs by symptom reported alone (in ascending order, compared to change in bowel habit)**	**Change in relative ranking (for moving from symptom-alone to symptom-multiple)**	**Rank of adjusted ORs by symptom reported with other symptoms (in ascending order, compared to other patients with multiple symptoms minus the symptom of interest)**
Abnormal mole	=	Abnormal mole
Breast lump	=	Breast lump
PMB	↓ –2	Rectal bleeding
Rectal bleeding	↑ +1	LUTS
LUTS	↑ +1	PMB
Haematuria	↓ –3	Lower abdominal pain
CIBH	↓ –4	Chest infection
Cough	↓ –2	Haemoptysis
Fatigue	↓ –3	Haematuria
Dyspnoea	↓ –3	Cough
Weight loss	↓ –4	CIBH
Any other symptom	↓ –6	Fatigue
Hoarseness	↓ –1	Dyspnoea
Chest infection	↑ +7	Hoarseness
Abdominal pain	↓ –1	Weight loss
Haemoptysis	↑ +8	Abdominal pain
Lower abdominal pain	↑ +11	Chest pain
Chest pain	↑ +1	Any other symptom
Back pain	=	Back pain
Neck lump	=	Neck lump

CIBH = change in bowel habit, LUTS: lower urinary tract symptoms, PMB = post-menopausal bleeding
Blue cells indicate symptoms that are relatively higher in rankings while yellow cells indicate symptoms that are relatively lower in rankings when comparing ORs associated with symptoms reported alone to symptoms reported with other symptoms.

Alternative parameterisation of advanced stage category as stage III–IV

Different parameterisation of stage at diagnosis was examined by defining late stage cases as those diagnosed at stages III or IV (stage IV in the main analysis) (n=7,997).

Reference group = patients with change in bowel habit. The non-shaded columns repeat data from the main analysis, presented here for ease of comparison.

	**Main analysis**			**Sensitivity analysis**		
**Symptom (seen alone)**	**N (%) with stage IV**	**Unadjusted OR (95% CI)[1]**	**Adjusted OR (95% CI)[2]**	**N (%) with stage III–IV**	**Unadjusted OR (95% CI)[1]**	**Adjusted OR (95% CI)[2]**
Abnormal mole	7 (1%)	0·04 (0·02–0·09)	0·17 (0·06–0·51)	38 (7%)	0·06 (0·04–0·09)	0·43 (0·20–0·93)
Breast lump	36 (3%)	0·11 (0·07–0·17)	0·20 (0·11–0·37)	163 (15%)	0·14 (0·10–0·19)	0·34 (0·21–0·55)
PMB	9 (4%)	0·12 (0·06–0·26)	0·40 (0·16–1·02)	33 (14%)	0·13 (0·08–0·21)	0·45 (0·24–0·85)
Rectal bleeding	28 (13%)	0·46 (0·27–0·77)	0·46 (0·27–0·78)	102 (47%)	0·70 (0·47–1·03)	0·66 (0·44–0·98)
LUTS	121 (15%)	0·54 (0·37–0·79)	0·56 (0·35–0·90)	325 (40%)	0·52 (0·38–0·72)	0·70 (0·47–1·05)
Haematuria	57 (18%)	0·65 (0·42–1·02)	0·79 (0·47–1·35)	98 (30%)	0·34 (0·23–0·49)	0·79 (0·50–1·24)
CIBH	46 (25%)	Ref	Ref	105 (56%)	Ref	Ref
Lower abdominal pain	18 (35%)	1·66 (0·85–3·22)	1·98 (1·00–3·94)	409 (47%)	0·68 (0·49–0·93)	0·91 (0·63–1·32)
Any other symptom	265 (30%)	1·32 (0·92–1·90)	1·27 (0·84–1·92)	36 (53%)	0·87 (0·50–1·52)	0·95 (0·41–2·23)
Abdominal pain	29 (33%)	1·47 (0·84–2·56)	1·45 (0·81–2·59)	31 (53%)	0·89 (0·49–1·60)	0·90 (0·49–1·67)
Hoarseness	21 (31%)	1·36 (0·74–2·51)	1·33 (0·57–3·10)	56 (63%)	1·31 (0·78–2·20)	1·52 (0·89–2·61)
Fatigue	18 (31%)	1·37 (0·72–2·62)	1·07 (0·54–2·10)	24 (47%)	0·69 (0·37–1·28)	0·90 (0·47–1·71)
Weight loss	27 (38%)	1·87 (1·04–3·35)	1·23 (0·66–2·28)	38 (54%)	0·89 (0·51–1·54)	0·78 (0·44–1·40)
Cough	72 (45%)	2·46 (1·56–3·88)	0·99 (0·59–1·65)	123 (76%)	2·50 (1·57–3·98)	1·42 (0·84–2·38)
Haemoptysis	33 (56%)	3·86 (2·09–7·13)	1·51 (0·78–2·92)	66 (61%)	1·21 (0·75–1·97)	0·75 (0·44–1·27)
Chest infection	34 (54%)	3·57 (1·96–6·48)	1·40 (0·73–2·66)	45 (71%)	1·93 (1·04–3·58)	1·07 (0·55–2·09)
Dyspnoea	52 (48%)	2·83 (1·71–4·68)	1·22 (0·70–2·12)	44 (75%)	2·26 (1·18–4·35)	1·25 (0·62–2·50)
Back pain	62 (58%)	4·19 (2·52–6·97)	3·19 (1·82–5·59)	76 (71%)	1·89 (1·14–3·14)	1·97 (1·13–3·43)
Chest pain	50 (60%)	4·61 (2·66–8·00)	2·12 (1·16–3·86)	66 (80%)	2·99 (1·63–5·49)	1·96 (1·03–3·75)
Neck lump	52 (80%)	12·17 (6·09–24·35)	5·62 (2·61–12·13)	63 (97%)	24·30 (5·77–102·28)	16·46 (3·76–72·10)
Joint Wald test	-	p<0·0010	p<0·0010	-	p<0·0010	p<0·0010

CIBH: change in bowel habit; LUTS: lower urinary tract symptoms; PMB: post-menopausal bleeding

[1] adjusted for symptoms

[2]adjusted for symptoms, sex, age group, ethnicity, IMD quintile, cancer diagnosis

Reference group = patients with multiple symptoms other than the symptom of interest. The non-shaded columns repeat data from the main analysis, presented here for ease of comparison.

	**Main analysis**			**Sensitivity analysis**		
**Symptom (seen with other symptoms)**	**N (%) with stage IV**	**Unadjusted OR (95% CI)[1]**	**Adjusted OR (95% CI)[2]**	**N (%) with stage III–IV**	**Unadjusted OR (95% CI)[1]**	**Adjusted OR (95% CI)[2]**
Abnormal mole	1 (5%)	0·06 (0·01–0·44)	0·44 (0·05–3·77)	3 (15%)	0·12 (0·04–0·42)	1·02 (0·26–4·09)
Breast lump	22 (12%)	0·14 (0·09–0·22)	0·45 (0·25–0·80)	68 (37%)	0·38 (0·27–0·53)	1·00 (0·63–1·58)
PMB	8 (13%)	0·18 (0·08–0·38)	0·79 (0·33–1·88)	19 (30%)	0·30 (0·17–0·52)	0·94 (0·48–1·84)
Rectal bleeding	52 (18%)	0·33 (0·23–0·46)	0·50 (0·35–0·74)	149 (53%)	0·74 (0·56–0·99)	0·78 (0·56–1·08)
LUTS	89 (27%)	0·44 (0·34–0·58)	0·77 (0·57–1·05)	69 (42%)	0·68 (0·53–0·86)	1·25 (0·86–1·82)
Haematuria	44 (27%)	0·53 (0·36–0·76)	0·98 (0·65–1·47)	157 (48%)	0·63 (0·45–0·87)	1·00 (0·75–1·34)
CIBH	190 (30%)	0·67 (0·54–0·83)	1·10 (0·85–1·41)	140 (60%)	1·30 (1·04–1·62)	1·39 (1·03–1·88)
Lower abdominal pain	65 (28%)	0·59 (0·43–0·80)	0·91 (0·66–1·26)	391 (62%)	1·45 (1·26–1·66)	1·60 (1·24–2·06)
Any other symptom	608 (39%)	0·94 (0·82–1·08)	1·55 (1·31–1·85)	213 (64%)	1·17 (0·62–2·22)	1·50 (1·15–1·96)
Abdominal pain	127 (38%)	0·89 (0·69–1·14)	1·37 (1·04–1·79)	957 (61%)	1·42 (1·08–1·87)	2·05 (1·71–2·46)
Hoarseness	30 (54%)	0·94 (0·54–1·62)	1·32 (0·71–2·44)	224 (73%)	1·23 (0·96–1·58)	1·57 (1·18–2·11)
Fatigue	152 (50%)	1·03 (0·80–1·32)	1·20 (0·92–1·56)	383 (75%)	1·13 (0·85–1·50)	1·64 (1·30–2·07)
Weight loss	260 (51%)	1·27 (1·04–1·55)	1·34 (1·08–1·65)	91 (76%)	1·66 (1·33–2·07)	1·09 (0·69–1·73)
Cough	289 (57%)	1·09 (0·86–1·37)	1·03 (0·80–1·31)	42 (75%)	2·13 (1·60–2·83)	1·35 (0·64–2·82)
Haemoptysis	64 (53%)	0·91 (0·62–1·34)	0·97 (0·66–1·44)	208 (82%)	1·79 (1·34–2·38)	1·39 (0·96–2·02)
Chest infection	142 (56%)	1·00 (0·75–1·34)	0·97 (0·72–1·30)	417 (82%)	1·60 (1·11–2·30)	1·80 (1·34–2·43)
Dyspnoea	237 (59%)	1·25 (0·99–1·58)	1·28 (1·00–1·64)	329 (81%)	1·13 (0·72–1·78)	1·67 (1·24–2·25)
Back pain	101 (62%)	2·05 (1·46–2·87)	2·76 (1·93–3·94)	123 (76%)	2·08 (1·42–3·04)	2·50 (1·67–3·72)
Chest pain	131 (62%)	1·42 (1·05–1·92)	1·45 (1·06–1·98)	173 (82%)	2·02 (1·38–2·96)	1·86 (1·26–2·74)
Neck lump	32 (80%)	3·91 (1·79–8·56)	3·55 (1·56–8·08)	38 (95%)	11·23 (2·69–46·92)	8·96 (2·09–38·36)
Joint Wald test	-	p<0·0010	p<0·0010	-	p<0·0010	p<0·0010

CIBH: change in bowel habit; LUTS: lower urinary tract symptoms; PMB: post-menopausal bleeding

[1] adjusted for symptoms

[2]adjusted for symptoms, sex, age group, ethnicity, IMD quintile, cancer diagnosis

Extreme case scenario for missing information on stage

An extreme missing not at random (MNAR) assumption was examined by assigning all patients with unknown stage (n=896) to stage IV (n=8,893). Reference group = patients with change in bowel habit. The non-shaded columns repeat data from the main analysis, presented here for ease of comparison.

	**Main analysis**			**Sensitivity analysis**		
**Symptom (seen alone)**	**N (%) with stage IV**	**Unadjusted OR (95% CI)[1]**	**Adjusted OR (95% CI)[2]**	**N (%) with stage IV**	**Unadjusted OR (95% CI)[1]**	**Adjusted OR (95% CI)[2]**
Abnormal mole	7 (1%)	0·04 (0·02–0·09)	0·17 (0·06–0·51)	61 (10%)	0·22 (0·15–0·33)	0·41 (0·22–0·75)
Breast lump	36 (3%)	0·11 (0·07–0·17)	0·20 (0·11–0·37)	149 (13%)	0·29 (0·21–0·41)	0·47 (0·29–0·76)
PMB	9 (4%)	0·12 (0·06–0·26)	0·40 (0·16–1·02)	22 (9%)	0·20 (0·12–0·34)	0·57 (0·29–1·14)
Rectal bleeding	28 (13%)	0·46 (0·27–0·77)	0·46 (0·27–0·78)	52 (22%)	0·56 (0·37–0·86)	0·60 (0·39–0·92)
LUTS	121 (15%)	0·54 (0·37–0·79)	0·56 (0·35–0·90)	220 (24%)	0·65 (0·47–0·90)	0·70 (0·47–1·04)
Haematuria	57 (18%)	0·65 (0·42–1·02)	0·79 (0·47–1·35)	103 (28%)	0·79 (0·55–1·14)	0·84 (0·54–1·31)
CIBH	46 (25%)	Ref	Ref	69 (33%)	Ref	Ref
Lower abdominal pain	18 (35%)	1·66 (0·85–3·22)	1·98 (1·00–3·94)	380 (38%)	1·26 (0·92–1·73)	1·23 (0·86–1·76)
Any other symptom	265 (30%)	1·32 (0·92–1·90)	1·27 (0·84–1·92)	27 (36%)	1·17 (0·67–2·03)	0·97 (0·44–2·11)
Abdominal pain	29 (33%)	1·47 (0·84–2·56)	1·45 (0·81–2·59)	23 (37%)	1·17 (0·65–2·10)	0·92 (0·50–1·71)
Hoarseness	21 (31%)	1·36 (0·74–2·51)	1·33 (0·57–3·10)	44 (42%)	1·49 (0·92–2·41)	1·55 (0·94–2·56)
Fatigue	18 (31%)	1·37 (0·72–2·62)	1·07 (0·54–2·10)	23 (41%)	1·41 (0·77–2·59)	1·76 (0·94–3·29)
Weight loss	27 (38%)	1·87 (1·04–3·35)	1·23 (0·66–2·28)	33 (43%)	1·52 (0·89–2·60)	1·05 (0·60–1·84)
Cough	72 (45%)	2·46 (1·56–3·88)	0·99 (0·59–1·65)	80 (47%)	1·82 (1·20–2·77)	0·94 (0·59–1·50)
Haemoptysis	33 (56%)	3·86 (2·09–7·13)	1·51 (0·78–2·92)	64 (53%)	2·32 (1·46–3·67)	1·14 (0·69–1·88)
Chest infection	34 (54%)	3·57 (1·96–6·48)	1·40 (0·73–2·66)	42 (59%)	2·94 (1·69–5·11)	1·35 (0·74–2·45)
Dyspnoea	52 (48%)	2·83 (1·71–4·68)	1·22 (0·70–2·12)	34 (57%)	2·65 (1·48–4·77)	1·29 (0·69–2·42)
Back pain	62 (58%)	4·19 (2·52–6·97)	3·19 (1·82–5·59)	69 (61%)	3·11 (1·94–4·99)	2·66 (1·59–4·46)
Chest pain	50 (60%)	4·61 (2·66–8·00)	2·12 (1·16–3·86)	55 (63%)	3·38 (2·01–5·68)	1·94 (1·11–3·40)
Neck lump	52 (80%)	12·17 (6·09–24·35)	5·62 (2·61–12·13)	62 (83%)	9·68 (4·98–18·79)	5·04 (2·44–10·42)
Joint Wald test	-	p<0·0010	p<0·0010	-	p<0·0010	p<0·0010

CIBH: change in bowel habit; LUTS: lower urinary tract symptoms; PMB: post-menopausal bleeding

[1] adjusted for symptoms

[2]adjusted for symptoms, sex, age group, ethnicity, IMD quintile, cancer diagnosis

Reference group = patients with multiple symptoms other than the symptom of interest. The non-shaded columns repeat data from the main analysis, presented here for ease of comparison.

	Main analysis			Sensitivity analysis		
**Symptom (seen with other symptoms)**	**N (%) with stage IV**	**Unadjusted OR (95% CI)[1]**	**Adjusted OR (95% CI)[2]**	**N (%) with stage IV**	**Unadjusted OR (95% CI)[1]**	**Adjusted OR (95% CI)[2]**
Abnormal mole	1 (5%)	0·06 (0·01–0·44)	0·44 (0·05–3·77)	2 (10%)	0·10 (0·02–0·43)	0·26 (0·06–1·19)
Breast lump	22 (12%)	0·14 (0·09–0·22)	0·45 (0·25–0·80)	54 (25%)	0·29 (0·21–0·41)	0·69 (0·44–1·08)
PMB	8 (13%)	0·18 (0·08–0·38)	0·79 (0·33–1·88)	13 (19%)	0·24 (0·13–0·45)	0·76 (0·38–1·54)
Rectal bleeding	52 (18%)	0·33 (0·23–0·46)	0·50 (0·35–0·74)	73 (24%)	0·39 (0·29–0·52)	0·55 (0·40–0·77)
LUTS	89 (27%)	0·44 (0·34–0·58)	0·77 (0·57–1·05)	60 (33%)	0·61 (0·44–0·85)	0·89 (0·62–1·27)
Haematuria	44 (27%)	0·53 (0·36–0·76)	0·98 (0·65–1·47)	125 (34%)	0·52 (0·41–0·67)	0·77 (0·59–1·02)
CIBH	190 (30%)	0·67 (0·54–0·83)	1·10 (0·85–1·41)	96 (36%)	0·71 (0·54–0·93)	0·99 (0·74–1·32)
Lower abdominal pain	65 (28%)	0·59 (0·43–0·80)	0·91 (0·66–1·26)	260 (37%)	0·75 (0·62–0·92)	1·03 (0·82–1·29)
Any other symptom	608 (39%)	0·94 (0·82–1·08)	1·55 (1·31–1·85)	171 (45%)	0·98 (0·78–1·24)	1·35 (1·06–1·73)
Abdominal pain	127 (38%)	0·89 (0·69–1·14)	1·37 (1·04–1·79)	822 (46%)	1·10 (0·97–1·25)	1·51 (1·29–1·77)
Hoarseness	30 (54%)	0·94 (0·54–1·62)	1·32 (0·71–2·44)	198 (56%)	1·13 (0·90–1·43)	1·19 (0·93–1·51)
Fatigue	152 (50%)	1·03 (0·80–1·32)	1·20 (0·92–1·56)	330 (57%)	1·34 (1·11–1·61)	1·35 (1·11–1·64)
Weight loss	260 (51%)	1·27 (1·04–1·55)	1·34 (1·08–1·65)	72 (56%)	0·94 (0·65–1·36)	0·95 (0·65–1·39)
Cough	289 (57%)	1·09 (0·86–1·37)	1·03 (0·80–1·31)	36 (58%)	0·99 (0·59–1·67)	1·19 (0·65–2·17)
Haemoptysis	64 (53%)	0·91 (0·62–1·34)	0·97 (0·66–1·44)	162 (59%)	1·03 (0·78–1·36)	0·97 (0·73–1·29)
Chest infection	142 (56%)	1·00 (0·75–1·34)	0·97 (0·72–1·30)	328 (60%)	1·13 (0·90–1·41)	1·04 (0·83–1·32)
Dyspnoea	237 (59%)	1·25 (0·99–1·58)	1·28 (1·00–1·64)	276 (62%)	1·30 (1·04–1·64)	1·27 (1·00–1·61)
Back pain	101 (62%)	2·05 (1·46–2·87)	2·76 (1·93–3·94)	122 (67%)	2·09 (1·52–2·89)	2·53 (1·81–3·54)
Chest pain	131 (62%)	1·42 (1·05–1·92)	1·45 (1·06–1·98)	144 (65%)	1·37 (1·02–1·85)	1·38 (1·02–1·88)
Neck lump	32 (80%)	3·91 (1·79–8·56)	3·55 (1·56–8·08)	38 (83%)	4·03 (1·86–8·71)	3·59 (1·61–7·97)
Joint Wald test	-	p<0·0010	p<0·0010	-	p<0·0010	p<0·0010

CIBH: change in bowel habit; LUTS: lower urinary tract symptoms; PMB: post-menopausal bleeding
[1] adjusted for symptoms
[2]adjusted for symptoms, sex, age group, ethnicity, IMD quintile, cancer diagnosis

Restricting analysis to patients who had a diagnostic interval (DI) of 0–60 days

Time to diagnosis may confound the association between presenting alarm symptoms and stage [1-3]; for example, prolonged intervals to help-seeking, referral/investigation, and diagnosis may be more common among patients with non-specific symptoms and those subsequently diagnosed with late stage disease. Of the study population used in the main analysis, there were 7,146/7,997 (89%) patients with complete information on the length of the diagnostic interval (DI); this sensitivity analysis was restricted to patients who were diagnosed within 60 days of symptomatic presentation (representing 65% (4,678/7,146) of those with complete DI information). The findings are presented in the blue shaded columns 5–7 of the tables below (n=4,678). Reference group = patients with change in bowel habit. The non-shaded columns repeat data from the main analysis, presented here for ease of comparison.

	**Main analysis**			**Sensitivity analysis**		
**Symptom (seen alone)**	**N (%) with stage IV**	**Unadjusted OR (95% CI)[1]**	**Adjusted OR (95% CI)[2]**	**N (%) with stage IV**	**Unadjusted OR (95% CI)[1]**	**Adjusted OR (95% CI)[2]**
Abnormal mole	7 (1%)	0·04 (0·02–0·09)	0·17 (0·06–0·51)	5 (1%)	0·03 (0·01–0·09)	0·15 (0·04–0·64)
Breast lump	36 (3%)	0·11 (0·07–0·17)	0·20 (0·11–0·37)	31 (3%)	0·09 (0·05–0·16)	0·34 (0·15–0·78)
PMB	9 (4%)	0·12 (0·06–0·26)	0·40 (0·16–1·02)	5 (3%)	0·09 (0·03–0·26)	0·26 (0·07–0·96)
Rectal bleeding	28 (13%)	0·46 (0·27–0·77)	0·46 (0·27–0·78)	13 (12%)	0·36 (0·17–0·75)	0·37 (0·18–0·77)
LUTS	121 (15%)	0·54 (0·37–0·79)	0·56 (0·35–0·90)	70 (19%)	0·62 (0·37–1·05)	0·48 (0·25–0·89)
Haematuria	57 (18%)	0·65 (0·42–1·02)	0·79 (0·47–1·35)	23 (15%)	0·48 (0·26–0·91)	0·46 (0·21–1·01)
CIBH	46 (25%)	Ref	Ref	26 (27%)	Ref	Ref
Lower abdominal pain	18 (35%)	1·66 (0·85–3·22)	1·98 (1·00–3·94)	137 (34%)	1·37 (0·84–2·25)	1·35 (0·77–2·38)
Any other symptom	265 (30%)	1·32 (0·92–1·90)	1·27 (0·84–1·92)	10 (31%)	1·21 (0·50–2·89)	1·79 (0·49–6·55)
Abdominal pain	29 (33%)	1·47 (0·84–2·56)	1·45 (0·81–2·59)	7 (26%)	0·93 (0·35–2·45)	0·79 (0·29–2·15)
Hoarseness	21 (31%)	1·36 (0·74–2·51)	1·33 (0·57–3·10)	18 (43%)	1·99 (0·93–4·25)	1·85 (0·84–4·07)
Fatigue	18 (31%)	1·37 (0·72–2·62)	1·07 (0·54–2·10)	9 (43%)	1·99 (0·75–5·28)	2·01 (0·73–5·54)
Weight loss	27 (38%)	1·87 (1·04–3·35)	1·23 (0·66–2·28)	15 (42%)	1·90 (0·85–4·23)	1·07 (0·45–2·51)
Cough	72 (45%)	2·46 (1·56–3·88)	0·99 (0·59–1·65)	31 (42%)	1·96 (1·03–3·74)	0·77 (0·37–1·60)
Haemoptysis	33 (56%)	3·86 (2·09–7·13)	1·51 (0·78–2·92)	33 (59%)	3·81 (1·90–7·65)	1·59 (0·74–3·44)
Chest infection	34 (54%)	3·57 (1·96–6·48)	1·40 (0·73–2·66)	20 (54%)	3·12 (1·42–6·87)	1·21 (0·51–2·87)
Dyspnoea	52 (48%)	2·83 (1·71–4·68)	1·22 (0·70–2·12)	23 (74%)	7·63 (3·03–19·19)	2·82 (1·06–7·51)
Back pain	62 (58%)	4·19 (2·52–6·97)	3·19 (1·82–5·59)	32 (62%)	4·25 (2·07–8·71)	2·61 (1·18–5·74)
Chest pain	50 (60%)	4·61 (2·66–8·00)	2·12 (1·16–3·86)	31 (57%)	3·58 (1·77–7·23)	1·59 (0·73–3·46)
Neck lump	52 (80%)	12·17 (6·09–24·35)	5·62 (2·61–12·13)	31 (79%)	10·28 (4·19–25·26)	7·13 (2·57–19·82)
Joint Wald test	-	p<0·0010	p<0·0010	-	p<0·0010	p<0·0010

CIBH: change in bowel habit; LUTS: lower urinary tract symptoms; PMB: post-menopausal bleeding

[1] adjusted for symptoms

2adjusted for symptoms, sex, age group, ethnicity, IMD quintile, cancer diagnosis

Reference group = patients with multiple symptoms other than the symptom of interest. The non-shaded columns repeat data from the main analysis, presented here for ease of comparison.

	Main analysis (Supplementary p5)			Sensitivity analysis		
**Symptom (seen with other symptoms)**	**N (%) with stage IV**	**Unadjusted OR (95% CI)[1]**	**Adjusted OR (95% CI)[2]**	**N (%) with stage IV**	**Unadjusted OR (95% CI)[1]**	**Adjusted OR (95% CI)[2]**
Abnormal mole	1 (5%)	0·06 (0·01–0·44)	0·44 (0·05–3·77)	0 (0%)	0·01 (0·00–0·03)	0·17 (0·04–0·67)
Breast lump	22 (12%)	0·14 (0·09–0·22)	0·45 (0·25–0·80)	19 (12%)	0·11 (0·06–0·18)	0·71 (0·34–1·51)
PMB	8 (13%)	0·18 (0·08–0·38)	0·79 (0·33–1·88)	5 (15%)	0·16 (0·06–0·41)	0·67 (0·20–2·28)
Rectal bleeding	52 (18%)	0·33 (0·23–0·46)	0·50 (0·35–0·74)	35 (22%)	0·44 (0·29–0·68)	0·71 (0·43–1·17)
LUTS	89 (27%)	0·44 (0·34–0·58)	0·77 (0·57–1·05)	21 (31%)	0·52 (0·30–0·91)	0·81 (0·44–1·50)
Haematuria	44 (27%)	0·53 (0·36–0·76)	0·98 (0·65–1·47)	48 (34%)	0·54 (0·37–0·80)	0·78 (0·51–1·21)
CIBH	190 (30%)	0·67 (0·54–0·83)	1·10 (0·85–1·41)	33 (30%)	0·57 (0·36–0·88)	0·82 (0·51–1·30)
Lower abdominal pain	65 (28%)	0·59 (0·43–0·80)	0·91 (0·66–1·26)	107 (30%)	0·52 (0·39–0·70)	0·87 (0·62–1·23)
Any other symptom	608 (39%)	0·94 (0·82–1·08)	1·55 (1·31–1·85)	87 (48%)	1·18 (0·85–1·64)	1·70 (1·18–2·44)
Abdominal pain	127 (38%)	0·89 (0·69–1·14)	1·37 (1·04–1·79)	385 (43%)	1·18 (0·98–1·42)	1·94 (1·53–2·47)
Hoarseness	30 (54%)	0·94 (0·54–1·62)	1·32 (0·71–2·44)	100 (58%)	1·26 (0·89–1·77)	1·38 (0·96–1·97)
Fatigue	152 (50%)	1·03 (0·80–1·32)	1·20 (0·92–1·56)	155 (54%)	1·28 (0·98–1·68)	1·35 (1·01–1·81)
Weight loss	260 (51%)	1·27 (1·04–1·55)	1·34 (1·08–1·65)	38 (59%)	1·07 (0·63–1·82)	1·16 (0·67–2·00)
Cough	289 (57%)	1·09 (0·86–1·37)	1·03 (0·80–1·31)	16 (59%)	0·96 (0·43–2·15)	1·55 (0·62–3·93)
Haemoptysis	64 (53%)	0·91 (0·62–1·34)	0·97 (0·66–1·44)	65 (56%)	0·84 (0·55–1·29)	0·82 (0·53–1·27)
Chest infection	142 (56%)	1·00 (0·75–1·34)	0·97 (0·72–1·30)	160 (60%)	1·14 (0·83–1·56)	1·09 (0·78–1·53)
Dyspnoea	237 (59%)	1·25 (0·99–1·58)	1·28 (1·00–1·64)	140 (64%)	1·43 (1·04–1·97)	1·54 (1·09–2·16)
Back pain	101 (62%)	2·05 (1·46–2·87)	2·76 (1·93–3·94)	53 (62%)	1·80 (1·13–2·89)	2·21 (1·35–3·63)
Chest pain	131 (62%)	1·42 (1·05–1·92)	1·45 (1·06–1·98)	69 (63%)	1·43 (0·93–2·19)	1·42 (0·91–2·21)
Neck lump	32 (80%)	3·91 (1·79–8·56)	3·55 (1·56–8·08)	21 (88%)	5·90 (1·74–20·02)	9·54 (2·59–35·12)
Joint Wald test	-	p<0·0010	p<0·0010	-	p<0·0010	p<0·0010

CIBH: change in bowel habit; LUTS: lower urinary tract symptoms; PMB: post-menopausal bleeding
[1] adjusted for symptoms
[2]adjusted for symptoms, sex, age group, ethnicity, IMD quintile, cancer diagnosis

References

1 Forrest LF, Adams J, Rubin G, White M. The role of receipt and timeliness of treatment in socioeconomic inequalities in lung cancer survival: population-based, data-linkage study. Thorax 2014; 70: 138–45. Available from: https://doi.org/10.1136/thoraxjnl-2014-205517
2 Neal RD, Tharmanathan P, France B, et al. Is increased time to diagnosis and treatment in symptomatic cancer associated with poorer outcomes? Systematic review. Br J Cancer 2015; 112: S92–107. Available from: https://doi.org/10.1038/bjc.2015.48
3 Tørring ML, Murchie P, Hamilton W, et al. Evidence of advanced stage colorectal cancer with longer diagnostic intervals: a pooled analysis of seven primary care cohorts comprising 11 720 patients in five countries. Br J Cancer 2017; 117 6: 1–10. Available from: https://doi.org/10.1038/bjc.2017.236

Adjustment for route to diagnosis

Diagnostic routes denote healthcare utilisation pathways before the diagnosis; cancers diagnosis in an emergency context is associated with poorer clinical outcomes which may confound the association between the studied symptoms and stage at diagnosis [1–3]. Therefore we additionally adjusted for route to diagnosis in the logistic regression model parameterised as one of five categories: 'Two-week-wait' referral (urgent referrals for suspected cancer from primary care to specialist hospital services); Elective referral (routine, non-urgent referrals); Emergency presentation; Secondary care (both inpatient and outpatient) routes; and Unknown route [1]. The resulting adjusted ORs are presented in the blue shaded column of the tables below (n=7,997).

Reference group = patients with change in bowel habit. The non-shaded columns repeat data from the main analysis, presented here for ease of comparison.

	**Main analysis**			**Sensitivity analysis**	
**Symptom (seen alone)**	**N (%) with stage IV**	**Unadjusted OR (95% CI)[1]**	**Adjusted OR (95% CI)[2]**	**N (%) with stage IV**	**Adjusted OR (95% CI)[2]**
Abnormal mole	7 (1%)	0·04 (0·02–0·09)	0·17 (0·06–0·51)	5 (1%)	0·14 (0·04–0·42)
Breast lump	36 (3%)	0·11 (0·07–0·17)	0·20 (0·11–0·37)	31 (3%)	0·20 (0·10–0·37)
PMB	9 (4%)	0·12 (0·06–0·26)	0·40 (0·16–1·02)	5 (3%)	0·38 (0·14–0·98)
Rectal bleeding	28 (13%)	0·46 (0·27–0·77)	0·46 (0·27–0·78)	13 (12%)	0·45 (0·26–0·76)
LUTS	121 (15%)	0·54 (0·37–0·79)	0·56 (0·35–0·90)	70 (19%)	0·50 (0·31–0·79)
Haematuria	57 (18%)	0·65 (0·42–1·02)	0·79 (0·47–1·35)	23 (15%)	0·68 (0·40–1·17)
CIBH	46 (25%)	Ref	Ref	26 (27%)	Ref
Lower abdominal pain	18 (35%)	1·66 (0·85–3·22)	1·98 (1·00–3·94)	137 (34%)	1·03 (0·68–1·58)
Any other symptom	265 (30%)	1·32 (0·92–1·90)	1·27 (0·84–1·92)	10 (31%)	1·45 (0·61–3·43)
Abdominal pain	29 (33%)	1·47 (0·84–2·56)	1·45 (0·81–2·59)	7 (26%)	1·07 (0·54–2·15)
Hoarseness	21 (31%)	1·36 (0·74–2·51)	1·33 (0·57–3·10)	18 (43%)	1·05 (0·58–1·90)
Fatigue	18 (31%)	1·37 (0·72–2·62)	1·07 (0·54–2·10)	9 (43%)	1·43 (0·70–2·92)
Weight loss	27 (38%)	1·87 (1·04–3·35)	1·23 (0·66–2·28)	15 (42%)	1·04 (0·55–1·96)
Cough	72 (45%)	2·46 (1·56–3·88)	0·99 (0·59–1·65)	31 (42%)	1·06 (0·63–1·78)
Haemoptysis	33 (56%)	3·86 (2·09–7·13)	1·51 (0·78–2·92)	33 (59%)	1·04 (0·59–1·85)
Chest infection	34 (54%)	3·57 (1·96–6·48)	1·40 (0·73–2·66)	20 (54%)	1·11 (0·57–2·16)
Dyspnoea	52 (48%)	2·83 (1·71–4·68)	1·22 (0·70–2·12)	23 (74%)	1·42 (0·72–2·79)
Back pain	62 (58%)	4·19 (2·52–6·97)	3·19 (1·82–5·59)	32 (62%)	2·39 (1·35–4·23)
Chest pain	50 (60%)	4·61 (2·66–8·00)	2·12 (1·16–3·86)	31 (57%)	1·84 (0·99–3·40)
Neck lump	52 (80%)	12·17 (6·09–24·35)	5·62 (2·61–12·13)	31 (79%)	5·40 (2·49–11·71)
Joint Wald test	-	p<0·0010	p<0·0010		p<0·0010

CIBH: change in bowel habit; LUTS: lower urinary tract symptoms; PMB: post-menopausal bleeding

[1] adjusted for symptoms
[2]adjusted for symptoms, sex, age group, ethnicity, IMD quintile, cancer diagnosis, and route to diagnosis

Reference group = patients with multiple symptoms other than the symptom of interest. The non-shaded columns repeat data from the main analysis, presented here for ease of comparison.

	**Main analysis**			**Sensitivity analysis**	
**Symptom (seen with other symptoms)**	**N (%) with stage IV**	**Unadjusted OR (95% CI)[1]**	**Adjusted OR (95% CI)[2]**	**N (%) with stage IV**	**Adjusted OR (95% CI)[2]**
Abnormal mole	1 (5%)	0·06 (0·01–0·44)	0·44 (0·05–3·77)	0 (0%)	0·44 (0·05–3·80)
Breast lump	22 (12%)	0·14 (0·09–0·22)	0·45 (0·25–0·80)	19 (12%)	0·49 (0·27–0·90)
PMB	8 (13%)	0·18 (0·08–0·38)	0·79 (0·33–1·88)	5 (15%)	0·76 (0·31–1·87)
Rectal bleeding	52 (18%)	0·33 (0·23–0·46)	0·50 (0·35–0·74)	35 (22%)	0·58 (0·39–0·85)
LUTS	89 (27%)	0·44 (0·34–0·58)	0·77 (0·57–1·05)	21 (31%)	0·90 (0·59–1·35)
Haematuria	44 (27%)	0·53 (0·36–0·76)	0·98 (0·65–1·47)	48 (34%)	0·76 (0·55–1·04)
CIBH	190 (30%)	0·67 (0·54–0·83)	1·10 (0·85–1·41)	33 (30%)	0·79 (0·57–1·10)
Lower abdominal pain	65 (28%)	0·59 (0·43–0·80)	0·91 (0·66–1·26)	107 (30%)	0·98 (0·75–1·27)
Any other symptom	608 (39%)	0·94 (0·82–1·08)	1·55 (1·31–1·85)	87 (48%)	1·12 (0·85–1·49)
Abdominal pain	127 (38%)	0·89 (0·69–1·14)	1·37 (1·04–1·79)	385 (43%)	1·19 (0·99–1·43)
Hoarseness	30 (54%)	0·94 (0·54–1·62)	1·32 (0·71–2·44)	100 (58%)	1·20 (0·91–1·57)
Fatigue	152 (50%)	1·03 (0·80–1·32)	1·20 (0·92–1·56)	155 (54%)	1·47 (1·18–1·82)
Weight loss	260 (51%)	1·27 (1·04–1·55)	1·34 (1·08–1·65)	38 (59%)	1·01 (0·67–1·51)
Cough	289 (57%)	1·09 (0·86–1·37)	1·03 (0·80–1·31)	16 (59%)	1·43 (0·76–2·68)
Haemoptysis	64 (53%)	0·91 (0·62–1·34)	0·97 (0·66–1·44)	65 (56%)	0·87 (0·64–1·18)
Chest infection	142 (56%)	1·00 (0·75–1·34)	0·97 (0·72–1·30)	160 (60%)	1·03 (0·80–1·33)
Dyspnoea	237 (59%)	1·25 (0·99–1·58)	1·28 (1·00–1·64)	140 (64%)	1·12 (0·87–1·45)
Back pain	101 (62%)	2·05 (1·46–2·87)	2·76 (1·93–3·94)	53 (62%)	2·49 (1·73–3·58)
Chest pain	131 (62%)	1·42 (1·05–1·92)	1·45 (1·06–1·98)	69 (63%)	1·30 (0·94–1·79)
Neck lump	32 (80%)	3·91 (1·79–8·56)	3·55 (1·56–8·08)	21 (88%)	3·62 (1·58–8·27)
Joint Wald test	-	p<0·0010	p<0·0010		p<0·0010

CIBH: change in bowel habit; LUTS: lower urinary tract symptoms; PMB: post-menopausal bleeding
[1] adjusted for symptoms
[2]adjusted for symptoms, sex, age group, ethnicity, IMD quintile, cancer diagnosis, and route to diagnosis

References

1 Elliss-Brookes L, McPhail S, Ives A, Greenslade M, Shelton J, Hiom S, et al. Routes to diagnosis for cancer – determining the patient journey using multiple routine data sets. Br J Cancer. 2012 Oct 20;107(8):1220–6. Available from: https://doi.org/10.1038/bjc.2012.408

2 Zhou Y, Abel GA, Hamilton W, Pritchard-Jones K, Gross CP, Walter FM, et al. Diagnosis of cancer as an emergency: a critical review of current evidence. Nat Rev Clin Oncol. 2017 Jan 11;14(1):45–56. Available from: https://doi.org/10.1038/nrclinonc.2016.155

3 McPhail S, Elliss-Brookes L, Shelton J, Ives A, Greenslade M, Vernon S, et al. Emergency presentation of cancer and short-term mortality. Br J Cancer. 2013;109(8):2027–34. Available from: https://doi.org/10.1038/bjc.2013.569

[End of Supplementary file]
